# Efficacy and Safety of Maxing Xianchang Su in the Treatment of Functional Constipation: A Randomized Controlled Trial

**DOI:** 10.1155/2021/3685440

**Published:** 2021-11-25

**Authors:** Mi Li, Lijuan Zhao, Li Ma, Wen Zhang, Hua Huang, Jun Wei, Jun Sun, Fenying Lu, Lijiang Ji

**Affiliations:** ^1^Changshu Hospital Affiliated to Nanjing University of Chinese Medicine, Changshu 215500, Jiangsu, China; ^2^Yueyang Hospital of Integrative Chinese and Western Medicine Affiliated to Shanghai University of Traditional Chinese Medicine, Shanghai 200437, China; ^3^Tianjin University of Traditional Chinese Medicine, Tianjin 301617, China; ^4^Jingjiang People's Hospital, Jingjiang 214500, Jiangsu, China; ^5^The No. 2 People's Hospital of Changshu, Changshu 215500, Jiangsu, China

## Abstract

**Background:**

Functional constipation **(**FC) is one of the prevalent gastrointestinal disorders that affect people of all ages. Long-term FC has significant effects on the quality of life of patients. Although commonly used drugs have reliable short-term effects, they are easily addictive and have side effects. Therefore, pursuing a convenient drug-food homogenous program is critical for FC patients. Maxing Xianchang Su is a functional food based on traditional Chinese medicine. To investigate the efficacy and safety of Maxing Xianchang Su in FC treatment, we conducted a randomized controlled trial.

**Methods:**

We carried out a prospective multicenter randomized parallel controlled study in three hospitals in Jiangsu Province, China, from January 2020 to March 2021, which included 206 FC patients. All patients were arbitrarily assigned into a treatment group and a control group at a ratio of 1 : 1; 103 cases in each group. The treatment group was given oral Maxing Xianchang Su, whereas the control group was treated with lactulose oral solution. The course of treatment was two weeks. The two groups of patients were evaluated after six weeks for symptom improvement before and after taking the drug. Furthermore, the safety of Maxing Xianchang Su was assessed.

**Results:**

Both groups of patients successfully completed the study without shedding cases. The effective rates of the treatment group and control group after two weeks were 90.6% and 67.0%, respectively. The treatment group had a better curative effect than the control group (*P* < 0.05). The symptom score of the two groups improved compared with that before the treatment. The difference between the two groups was statistically significant (*P* < 0.05). During the treatment process, neither group experienced abnormal changes in blood lipid, blood glucose, routine hematuria, or liver and kidney functions. There were no adverse reactions in both groups.

**Conclusion:**

Maxing Xianchang Su has a positive effect on FC treatment with reliable mid-term effect and a high level of safety.

## 1. Introduction

Functional constipation (FC) refers to constipation without an organic etiology. Symptoms include difficulty in defecation and reduced stool frequency, which are often accompanied by abdominal distension and pain [1]. Functional constipation has a more complicated mechanism that involves emotions, diet, pelvic floor function, and intestinal flora [2]. The therapy of FC focuses on symptomatic treatment with the primary aim of restoring normal intestinal functions, maintaining a healthy intestinal microecology, and improving the quality of life of patients. Drug and nondrug therapies are currently used to treat FC. Drug therapy includes fiber supplements, osmotic and stimulant laxatives, stool softeners, enemas, and other drugs [3]. Although the drugs have reliable short-term effects, they are typically dependent. Furthermore, some of the drugs have severe side effects [4]. Nondrug treatments include dietary adjustment, emotional conditioning, appropriate exercise, regular defecation, and other beneficial habits that make the stool smooth and improve the symptoms [5]. However, the efficacy is uncertain and the therapy cycle is long.

Thousands of years of development and clinical practice of traditional Chinese medicine have revealed that several Chinese herbal medicines provide excellent benefits in the treatment of FC [6]. However, the clinical use and promotion of the traditional Chinese medicinal decoction are severely restricted because of its inconvenient and poor taste. A growing number of scholars are advocating the use of functional foods to solve some chronic diseases [7]. At present, some probiotics-based functional foods are being used in the treatment of FC; however, the clinical efficacy is insignificant [8].

Maxing Xianchang Su, a functional and edible food with the same medicinal effects, is developed by Changshu Hospital Affiliated to Nanjing University of Chinese Medicine in China based on traditional Chinese medicine. The ingredients of Maxing Xianchang Su include Huo Ma Ren (*Semen Cannabis*), Xing Ren (*Semen Armeniacae Amarum*), Chen pi (*Pericarpium Citri Reticulatae*), lard, wheat dietary fiber, polydextrose, and xylooligosaccharide. Although some clinical studies have shown that Maxing Xianchang Su is beneficial to FC patients [9], its properties and long-term efficacy remain unclear. Moreover, it is not known whether the sugar and oil contained in Maxing Xianchang Su affect blood lipid and glucose levels in FC patients. Therefore, this study evaluated the efficacy and safety of Maxing Xianchang Su on FC using a prospective multicenter randomized parallel controlled study in three hospitals in Jiangsu Province, China.

## 2. Methods

### 2.1. Study Design

**Figure 1:**
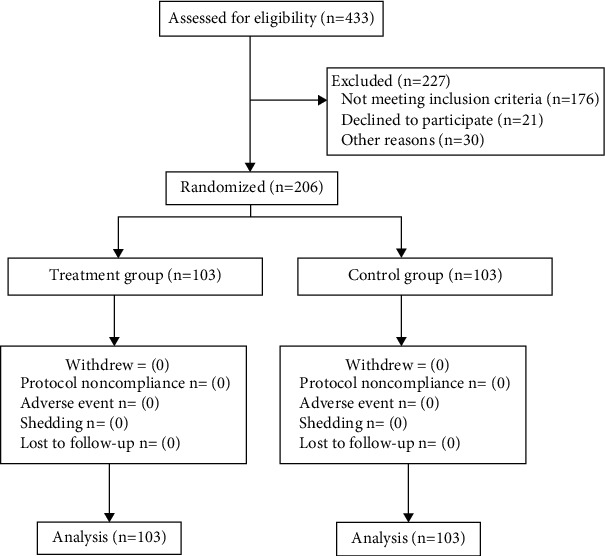
Flow diagram.

This study was conducted in three hospitals in Suzhou, including Changshu Hospital Affiliated to Nanjing University of Chinese Medicine, Jingjiang People's Hospital of Jiangsu Province, and Second People's Hospital of Changshu City, Jiangsu Province. Patients were recruited through outpatient clinics and networks. All participants were required to continue their current lives, and all patients had a 2-week observation period before being formally included in the study to eliminate the impact of special dietary components and living habits on the study's outcomes. We shall not change the dietary habits or lifestyles of patients throughout this time to prevent influencing the study's results. The patients' clinical manifestations match the study's criteria and are included in the study when the observation period ended. Patients who met the inclusion criterion were arbitrarily divided into two groups: treatment and control. Both groups were treated for two weeks and monitored for another six weeks. During the study, patients were not allowed to take any food or drugs that would have induced bowel movement, and their defecation and stool characteristics were recorded. The investigator evaluated the efficacy (total effective rate) at the time of enrollment (baseline), at the second week (after treatment), and at the eighth week (follow-up) to evaluate the patient's stool symptom score, blood lipids, blood glucose, hematuria routine, and liver and kidney functions.  Figure 1 demonstrates the research flow diagram.

### 2.2. Ethics and Registration

This research followed the latest consolidated standards of reporting trials (CONSORT 2017) and standard protocol items: recommendations for interventional trials (SPIRIT) 2013 statement (SPIRIT checklist, see Table S1). Furthermore, the protocol was conducted in accordance with the declaration of Helsinki and ethical guidelines for clinical research. The study was reviewed and approved by the ethics committee of Changshu Hospital Affiliated to Nanjing University of Chinese Medicine (ethics number: 20190053). Finally, each patient signed a written informed consent form.

### 2.3. Study Patients

All participants were diagnosed with primary FC. The diagnostic, inclusion, and exclusion criteria were as follows.

#### 2.3.1. Diagnostic Criteria

The diagnostic criteria for FC were based on Roman III criteria [10]. The specific details are as follows: (1) two or more of the following symptoms had to be met: ① laborious defecation at least 25% of the time; ② lumpy defecation or hard stools at least 25% of the time (Bristol Stool Form Scale 1 or 2 [11], see Table 1); ③ a sense of incomplete defecation at least 25% of the time; ④ an anorectal obstruction or feeling of obstruction at least 25% of the time; ⑤ defecation needed manual assistance, such as assisting defecation with fingers or pelvic floor support, at least 25% of the time; ⑥ spontaneous defecation was less than three times a week. (2) Loose stools rarely occurred when laxatives were not used. (3) Did not meet the diagnostic criteria for irritable bowel syndrome with predominant constipation (IBS-C).

#### 2.3.2. Inclusion Criteria

  ① Organic factors, drugs, and other secondary factors were excluded from the diagnosis of FC
  ② The age ranged from 18 to 80 years old
  ③ No medical conditions such as diabetes, hyperlipidemia, or visceral tumors
  ④ No other drugs and methods for constipation were used within one month before taking this product
  ⑤ The patients voluntarily participated in the study and signed an informed consent form

#### 2.3.3. Exclusion Criteria

  ① Patients with a history of abdominal, rectal, or anal surgery
  ② Patients who were unable to quit the drug because of constipation caused by the drug
  ③ Persons who were allergic to the drugs and food used in this study
  ④ Patients with poor compliance or were participating in other clinical studies at the same time

### 2.4. Sample Size

The total effective rate of the main efficacy indicators was used to estimate the sample size for this study. Preliminary results indicated that the total effective rates of Maxing Xianchang Su and lactulose oral liquid was 91% and 64%, respectively. For sample size estimation, PASS15.0 was used. The optimal design was adopted, in which *α* = 0.05, test efficiency = 0.80, boundary value = 0.10, and the number of cases in the treatment group: the number of cases in the control group = 1 : 1. The calculated sample size for the two groups was 184 cases. However, after considering the 10% abscission rate, a total of 206 cases were finally included, with 103 cases in each group.

### 2.5. Randomization and Blinding

This study used stratified randomization for the enrolled 206 patients and for each center (70 in Changshu Hospital Affiliated to Nanjing University of Chinese Medicine, 70 in Jingjiang People's Hospital in Jiangsu Province, 66 in Second People's Hospital in Changshu City, Jiangsu Province). Excel 2013 software was used to generate arbitrary numbers and divide them randomly into two groups in a 1 : 1 ratio for the complete randomization method. The grouping was sealed in an opaque envelope with a number inscribed on it, and the patients were asked to pick an envelope arbitrarily to obtain the corresponding random number to finish the grouping. Throughout the study, the research assistant was responsible for screening, recruiting, and assigning random numbers to the participants. Random assignment was done by doctors who were not involved in treatment decisions. The patients and the principal investigator were aware of the distribution results because the appearance and taste of the drugs taken differed in the treatment and control groups. However, data statisticians and analysts were unaware of the distribution plan.

### 2.6. Therapeutic Interventions

The treatment group received Maxing Xianchang Su (strictly produced in accordance with the standards of Good Manufactory Practice (GMP) and Chinese Pharmacopoeia 2010 by the Changshu Hospital Affiliated to Nanjing University of Chinese Medicine) orally [12]. The ingredients are shown in Table 2. For two weeks, the patients were instructed to chew one piece (25 g/piece) and drink 300 mL of water after eating three times a day.

The formula of Maxing Xianchang Su is as follows: polyglucose 34 g, expanded wheat flour 93 g, wheat fiber particles 37 g, lard 150 g, sugar 125 g, xylooligosaccharide 13 g, almond 50 g, orange peel 30 g, hemp seed 50 g, egg 150 g, leavening agent 4 g (baking soda 2 g and ammonium bicarbonate 2 g). Bake at this temperature (surface temperature: 150°C and bottom temperature: 140°C) for 30 minutes [9].

The control group took oral lactulose oral liquid (Sichuan Jianneng Pharmaceutical Co., Ltd., Sichuan, China, H20103621; 100 ml: 66.7 g). The participants were instructed to take 15 mL twice in a day for two weeks.

### 2.7. Outcome Indicators

#### 2.7.1. Primary Outcome Indicators

Total effective rate: the efficacy was evaluated according to the clinical efficacy calculation method (efficacy index = (posttreatment score pretreatment score)/pretreatment score × 100%) [13, 14]. The clinical efficacy was evaluated according to the “Guiding principles for clinical research of new traditional Chinese medicine (trial)” [15]. The clinical curative effect was divided into recovery, markedly effective, effective, and ineffective. Recovery: the discomfort symptoms of constipation disappeared, and the reduction in symptom score was more than 95%. Markedly effective: the clinical symptoms were significantly improved, and the symptom score reduction ranged from 70% to 95%. Effective: the clinical symptoms were alleviated, and the symptom score reduction ranged from 30% to 70%. Ineffective: no improvement or aggravation of the clinical symptoms, and the reduction in the symptom score was less than 30%. The total effective rate = (the number of people cured + the number of people in the markedly effective category + the number of people in the effective category)/total number of people × 100%. The efficacy index of this study was the total score of stool symptoms.

#### 2.7.2. Secondary Outcome Indicators

The stool symptom score refers to the FC symptom quantification scoring standard established in the 2017 “Consensus on the diagnosis and treatment of functional constipation integrated traditional Chinese and western medicine” [1] (Table 3).

### 2.8. Safety Evaluation

Safety evaluation included the general conditions of the participants, the incidence of adverse events (such as rashes, abdominal pain, abdominal distension, diarrhea, dizziness, and other uncomfortable symptoms during the study period), and laboratory indicators (such as blood routine, urine routine, and liver and kidney functions). As Maxing Xianchang Su contains sugar and oil, we focused on the changes in blood sugar and blood lipids before and after the treatment. Hematological indices were obtained before and after the treatment and early in the morning at three different time points during the follow-up period. The venous blood was tested while the patients were fasting.

### 2.9. Statistical Analysis

Statistical analyses were performed using SPSS 21.0 software. The chi-square test was used on the enumeration data, whereas the mean ± standard deviation ($\bar{x}$ ± *s*) was used on the measurement data. The independent sample *t*-test was used for the normal distribution, while the nonparametric test was used for the skewed distribution. The difference was considered to be statistically significant when *P* < 0.05.

## 3. Results

### 3.1. Baseline Characteristics

A total of 206 FC patients were included in this study. None of the participants withdrew from the study. The treatment group and the control group each had 103 cases. There was no significant difference in baseline characteristics between the two groups (*P* > 0.05; Table 4).

### 3.2. Evaluation of the Outcome

#### 3.2.1. The Total Effective Rates between the Two Groups

The total effective rates after two weeks in the treatment group and control group were 90.6% and 67.0%, respectively. The treatment group had a better effective rate than the control group, and the difference was statistically significant (*P* < 0.05; Table 5).

#### 3.2.2. Symptom Scores in the Two Groups before and after Treatment

There was no significant difference in defecation frequency, defecation time, difficult defecation, abdominal distension, and stool character score between the two groups before treatment (*P* > 0.05). The scores of defecation symptoms in the two groups of patients improved after the treatment, and the treatment group was better than the control group; the difference was statistically significant (*P* < 0.05; Tables 6 and 7).

#### 3.2.3. Symptom Scores between the Two Groups of Patients after Six Weeks of Follow-up

After six weeks of follow-up, the defecation symptom scores of the two groups of patients improved more considerably than at the end of the treatment. The treatment group was better than the control group, with a statistically significant difference (*P* < 0.05) as shown in Table 8.

#### 3.2.4. Comparison of Symptom Scores between Male and Female Patients in Treatment Group before and after Treatment

There were no significant differences in frequency, defecation time, defecation difficulty, abdominal distension, and stool traits between the male and female patients before the treatment (*P* > 0.05). After the treatment, there was no significant difference between the male and female patients in defecation frequency, defecation time, and defecation traits (*P* > 0.05). In terms of defecation difficulty and abdominal distension, the male scores were higher than the female scores (*P* < 0.05), as shown in Tables 9 and 10.

#### 3.2.5. Comparison of Symptom Scores between Male and Female Patients in the Treatment Group after 6 Weeks of Follow-Up

After a 6-week follow-up, there were no significant differences between the male and female patients in defecation frequency, defecation time, defecation difficulty, abdominal distension, and defecation traits (*P* > 0.05), as shown in Table 11.

#### 3.2.6. Blood Glucose and Blood Lipids Levels in the Two Groups of Patients before and after Treatment

There were no abnormal fluctuations in the blood lipids and blood glucose in either group in the 0 week, 2^nd^ week, or 8^th^ week. There was no statistical significance between the treatment and control groups (*P* > 0.05), as shown in Table 12.

#### 3.2.7. Safety and Adverse Events

Patients in both groups did not suffer any discomfort during the observation period of the study. There were no abnormalities in blood routine, urine routine, liver function, and kidney function indices of the two groups of patients before and after the treatment. Detailed data were presented in Table S2.

## 4. Discussion

Functional constipation (FC) is a common gastrointestinal disorder that is clinically characterized by difficulty in defecating, prolonged defecation time, and a low defecation frequency [16]. It has significant effects on the quality of life of patients, poses a risk to physical and mental health, and has a substantial impact on the occurrence and development of many diseases [17]. An epidemiological survey in China shows that the prevalence of constipation in Chinese adults is between 3% and 17% [18]. Women outnumber men and the prevalence increases with age [19]. Studies have shown that inert intestinal motility, insufficient dietary fiber intake, intestinal microecological imbalance, and emotional factors are the main causes of FC [16]. Dietary structure adjustment, drug intervention, and intestinal microecological adjustment are currently among the main methods for FC treatment [20]. Western medicine gives symptomatic treatment to patients using drugs that improve gastrointestinal motility and promote excretion. Although the initial effect is strong, the long-term maintenance effect is weak, and there are many adverse reactions [21].

In traditional Chinese medicine, FC is classified as “intestinal dryness” and “constipation.” The main pathogenesis is intestinal conduction failure and Tongjiang imbalance [22]. Maxing Xianchang Su is a functional food developed based on Maziren Pill, which is a famous prescription from the “Shanghanlun” (a medical book written by Zhang Zhongjing, a renowned physician in the Eastern Han Dynasty) that acts as a laxative and moistens the intestines [23]. Maxing Xianchang Su selects the main ingredients of Ma Zi Ren Pills, Huo Ma Ren (*Semen Cannabis*), Xing Ren (*Semen Armeniacae Amarum*), polydextrose, puffed wheat flour, wheat fiber particles, lard, and sucrose. It has greater relaxing medicinal properties than Maziren Pills, is not restricted by TCM syndromes, and is used universally [9, 24]. Huo Ma Ren (*Semen Cannabis*) and Xing Ren (*Semen Armeniacae Amarum*) are both Chinese medicinal ingredients from the same sources of medicine and food. They are rich in oil with approximately 35% in Huo Ma Ren (*Semen Cannabis*) and 40%∼50% in Xing Ren (*Semen Armeniacae Amarum*) [25], which can lubricate the intestines and help in defecating. Hemp seed contains unsaturated fatty acids and hemp seed oil, which can improve the intestinal microecological environment and optimize the structure of the intestinal flora [25]. Amygdalin in almonds and naringin in tangerine peel, can cause colonic smooth muscle to contract spontaneously [26, 27]. Polydextrose is a commonly used water-soluble dietary fiber (SDF) with a complex mechanism for treating FC, which may be because it can directly enter the intestine and produce a large number of short chain fatty acids (SCFAs). When the SCFAs are utilized by the intestinal beneficial bacteria, they may stimulate intestinal peristalsis and increase fecal water content and volume, promoting fecal excretion [28]. Clinical studies have confirmed that polydextrose can improve defecation frequency and fecal properties, and 12 g/day is the optimal dose for curative effect [29, 30]. Wheat fiber granules contain water-insoluble dietary fiber (IDF). The IDF: SDF in the Maxing Xianchang Su is 17.2%:9.5% (Qingdao Kechuang Quality Inspection Co., Ltd.). The proportions are reasonable, and the total dietary fiber content is over 26.7%, resulting in a satisfactory defecation effect. Lactulose is a disaccharide compound that changes the osmotic pressure of the intestinal tract through physical action. It has permeability for disaccharides, which preserves water and electrolytes in the intestinal tract while softening stool and guiding excretion. It is a regularly prescribed medication for constipation [31, 32].

In this study, the results showed that the bowel symptoms of the two groups of patients improved after two weeks of treatment. The treatment group had better symptom scores and effective rates than the control group (*P* < 0.05). The improvement of each symptom score of the treatment group remained significantly better than that of the control group after six weeks of follow-up (*P* < 0.05). The results revealed no abnormality in blood glucose and blood lipids in the patients before and after the treatment, and the difference between the groups was not statistically significant (*P* > 0.05). To investigate whether Maxing Xianchang Su had a difference in efficacy between men and women, we compared the treatment results by gender and discovered that there was no statistical difference in the stool symptom scores of men and women before treatment (*P* > 0.05), indicating that the sexes were not comparable. Men and women had no significant differences in defecation frequency, defecation time, or defecation traits after treatment (*P* > 0.05). Males scored higher than females in terms of defecation difficulty and abdominal distension (*P* < 0.05), i.e., Maxing Xianchang Su is more effective in treating bowel dysfunction and abdominal distension in women than in men. There was no significant difference in defecation frequency, defecation time, defecation difficulty, abdominal distension, or defecation features between the male and female patients at the 6-week follow-up (*P* > 0.05), indicating that the efficacy of Maxing Xianchang Su was not connected to gender in the long run. Females had a higher prevalence of functional constipation, according to studies [33], which is consistent with the features seen in this study (125/206). Some symptoms (difficulty defecating and stomach distension) differed between males and females after treatment. We cannot say whether this is related to gender differences because of the small sample size. There is no doubt that Maxing Xianchang Su is beneficial for functional constipation in both male and female patients based on the outcomes of the follow-up. In future trials, it will be examined whether Maxing Xianchang Su has different efficacies in men and women. The results of this study show that Maxing Xianchang Su can effectively improve the stool frequency and traits of FC patients. The curative effect is safe and reliable, and it does not affect the blood sugar and blood lipids levels in the patients. Furthermore, the patients did not experience liver and kidney damage, abdominal pain, diarrhea, dizziness, or other adverse reactions while using Maxing Xianchang Su. Moreover, Maxing Xianchang Su is tasty, portable, and easy to use. These findings led to a preliminary conclusion: Maxing Xianchang Su improves the patient's defecation condition considerably, making it a convenient, effective, and safe new treatment option for FC.

This study has the following shortcomings: the used intervention method could not blind the physicians and patients, which may result in biased findings. Furthermore, this study did not conduct a stratified analysis of the included population, such as the elderly, young people, etc., making it impossible to clarify the effect of Maxing Xianchang Su on different groups of people.

To summarize, Maxing Xianchang Su has a positive effect on FC with a reliable mid-term effect. Additionally, it does not adversely affect the blood lipids, blood sugar, liver and kidney functions, and blood and urine routines, and it has a high level of safety. Therefore, it is a safe and effective method to treat FC.

## Figures and Tables

**Table 1:** Bristol stool scale.

Classification	Fecal property	Score
Level 1	Separate hard lumps, like nuts	3
Level 2	Sausage shaped but lumpy	2
Level 3	Like a sausage or snake but with cracks on its surface	1
Level 4	Like a sausage or snake, smooth and soft	0
Level 5	Soft blobs with clear cut edges	0
Level 6	Fluffy pieces with ragged edges, a mushy stool	0
Level 7	Watery stool, no solid block (completely liquid)	0

**Table 2:** Ingredients of Maxing Xianchang Su.

Ingredients	Composition (%)	Basic formulation (g)	g/piece	g/day
Huo Ma Ren (*Semen Cannabis*)	7	7	1.75	5.25
Xing Ren (*Semen Armeniacae Amarum*)	4	4	1	3
Chen pi (*Pericarpium Citri Reticulatae*)	3	3	0.75	2.25
Wheat dietary fiber	7	7	1.75	5.25
Puffed wheat flour	13	13	3.25	9.75
Polydextrose	7	7	1.75	5.25
Sucrose	12	12	3	9
Xylo-oligosaccharide	7	7	1.75	5.25
Lard	18	18	4.5	13.5
Egg	23	23	5.75	12.75

**Table 3:** Quantitative scoring criteria for functional constipation symptoms.

Entry	Criteria			
Defecation frequency (d/times)	1∼2	3	4∼5	>5
Defecation time (min/times)	<10	10∼15	15∼25	>25
Difficult defecation	Never	Seldom	Sometimes	Often
Abdominal distension	Never	Seldom	Sometimes	Often
Bristol stool scale	4∼7	3	2	1
Score	0	1	2	3

**Table 4:** Comparison of basic data between the treatment and control groups ($\bar{x}$ ± *s*).

Group	Cases	Sex		Ages	Course (years)
		Men	Women		
Treatment group	103	39	64	49.96 ± 19.80	13.49 ± 8.42
Control group	103	42	61	49.76 ± 20.33	14.28 ± 8.37
*P* value		0.62		0.943	0.211

**Table 5:** Comparison of total effective rates between the treatment and control groups.

Group	Cure	Markedly effective	Effective	Ineffective	Total effective rate (%)
Treatment group	22	47	24	10	90.6^*∗*^
Control group	12	18	39	34	67.0

Note: ^*∗*^*P* < 0.05 when compared to the control group.

**Table 6:** Comparison of changes in the symptom scores before and after treatment in the treatment and control groups ($\bar{x}$ ± *s*).

Group	Defecation frequency		Defecation time		Difficult defecation	
	0 weeks	2^nd^ week	0 weeks	2^nd^ week	0 weeks	2^nd^ week
Treatment group	1.27 ± 0.38	0.38 ± 0.11^*∗*^	1.47 ± 0.67	0.45 ± 0.15^*∗*^	1.94 ± 0.80	0.57 ± 0.19^*∗*^
Control group	1.27 ± 0.37	0.76 ± 0.17^*∗*^	1.45 ± 0.66	0.89 ± 0.18^*∗*^	1.97 ± 0.76	1.29 ± 0.40^*∗*^
*P* value	0.925	<0.001	0.836	<0.001	0.793	<0.001

Note: ^*∗*^*P* < 0.05 when compared to before treatment in the groups.

**Table 7:** The comparison of changes in symptom scores before and after treatment in the treatment and control groups ($\bar{x}$ ± *s*).

Group	Abdominal distension		Stool character score	
	0 weeks	2 weeks	0 weeks	2 weeks
Treatment group	2.19 ± 0.81	0.42 ± 0.18^*∗*^	1.91 ± 0.76	0.59 ± 0.16^*∗*^
Control group	2.11 ± 0.87	1.08 ± 0.22^*∗*^	1.91 ± 0.72	1.08 ± 0.26^*∗*^
*P* value	0.512	<0.001	0.963	<0.001

Note: ^*∗*^*P* < 0.05 when compared with before treatment in the groups.

**Table 8:** Comparison of follow-up symptom scores between the treatment and the control groups ($\bar{x}$ ± *s*).

Group	Defecation frequency	Defecation time	Difficult defecation	Abdominal distension	Stool character score
Treatment group	0.37 ± 0.49	0.29 ± 0.45	0.43 ± 0.52	0.31 ± 0.47	0.46 ± 0.62
Control group	0.80 ± 0.66	0.66 ± 0.50	1.19 ± 0.57	1.00 ± 0.66	1.02 ± 0.72
*P* value	≤0.01	≤0.01	≤0.01	≤0.01	≤0.01

**Table 9:** Comparison of symptom scores between male and female patients in treatment group before and after treatment ($\bar{x}$ ± *s*).

Groups	Defecation frequency		Defecation time		Difficult defecation	
	0 weeks	2^nd^ week	0 weeks	2^nd^ week	0 weeks	2^nd^ week
Male	1.18 ± 0.76	0.41 ± 0.50	1.38 ± 0.71	0.51 ± 0.64	1.97 ± 0.78	0.79 ± 0.73
Female	1.33 ± 0.64	0.33 ± 0.51	1.55 ± 0.62	0.39 ± 0.49	1.91 ± 0.83	0.41 ± 0.61
*P* value	0.290	0.423	0.224	0.312	0.680	0.005

**Table 10:** Comparison of symptom scores of male and female patients in the treatment group before and after treatment ($\bar{x}$ ± *s*).

Groups	Abdominal distension		Defecation traits	
	0 weeks	2^nd^ week	0 weeks	2^nd^ week
Male	2.08 ± 0.90	0.62 ± 0.75	1.87 ± 0.66	0.69 ± 1.00
Female	2.23 ± 0.75	0.28 ± 0.58	1.95 ± 0.72	0.52 ± 0.71
*P* value	0.341	0.020	0.568	0.340

**Table 11:** The comparison of symptom scores between male and female patients in the treatment group during follow-up ($\bar{x}$ ± *s*).

Groups	Defecation frequency	Defecation time	Difficult defecation	Abdominal distension	Defecation traits
Male	0.28 ± 0.46	0.36 ± 0.49	0.54 ± 0.51	0.38 ± 0.49	0.51 ± 0.68
Female	0.28 ± 0.45	0.36 ± 0.48	0.36 ± 0.52	0.25 ± 0.44	0.42 ± 0.56
*P* value	0.993	0.997	0.088	0.165	0.463

**Table 12:** The comparison of blood glucose and blood lipids contents between the treatment and control groups before and after treatment ($\bar{x}$ ± *s*).

Variable	Treatment group	Control group	*P* value
Total cholesterol (mmol/L)			
0 weeks	3.18 ± 1.21	3.52 ± 1.74	0.10
2^nd^ week	3.31 ± 2.07	3.59 ± 1.86	0.31
8^th^ week	3.28 ± 1.76	3.39 ± 2.46	0.71
Triglyceride (mmol/L)			
0 weeks	1.13 ± 0.94	1.06 ± 0.84	0.57
2^nd^ week	1.21 ± 0.87	1.18 ± 0.79	0.80
8^th^ week	1.09 ± 0.98	1.12 ± 0.88	0.82
Low density lipoprotein (mmol/L)			
0 weeks	1.96 ± 1.51	1.82 ± 1.73	0.54
2^nd^ week	2.23 ± 1.62	2.01 ± 1.82	0.36
8^th^ week	2.11 ± 1.71	2.19 ± 1.64	0.73
Blood glucose (mmol/L)			
0 weeks	4.54 ± 1.93	4.79 ± 2.01	0.36
2^nd^ week	4.77 ± 1.74	4.97 ± 1.95	0.44
8^th^ week	5.05 ± 1.88	4.83 ± 2.17	0.44

## Data Availability

The data for this study can be obtained from the corresponding author.

## Abbreviations

FC: Functional constipation
IBS-C: Irritable bowel syndrome with predominant constipation
GMP: Good Manufactory Practice
SCFAs: Short chain fatty acids
IDF: Insoluble dietary fiber.

## Supplementary Materials

Supplementary Materials Table S1: the SPIRIT checklist. Table S2: detailed data of security indicators.
