# Supplementary material for: Efficacy and Safety of Maxing Xianchang Su in the Treatment of Functional Constipation: A Randomized Controlled Trial
Source: Evid Based Complement Alternat Med. 2021 Nov 25;2021:3685440. doi: 10.1155/2021/3685440 (PMC8639258; doi:10.1155/2021/3685440)
Supplement: Supplementary Materials — Table S1: the SPIRIT checklist. Table S2: detailed data of security indicators. [file 3685440.f1.zip › 3685440.f1/Table S2Detailed data of security indicators..docx]

Table S2：Detailed data of security indicators.

| Indices | Variable | Treatment group | Control group | *P* value |
| --- | --- | --- | --- | --- |
| Blood routine | Hb (g/L) |  |  |  |
|  | 0 weeks | 134.32±18.21 | 129.89±21.74 | 0.11 |
|  | 2^nd^ week | 136.51±21.07 | 132.12±22.37 | 0.15 |
|  | 8^th^ week | 134.98±20.76 | 131.61±23.46 | 0.27 |
|  | RBC (10^12^/L) |  |  |  |
|  | 0 weeks | 4.54±1.91 | 4.81±2.01 | 0.32 |
|  | 2^nd^ week | 4.77±1.71 | 4.92±1.93 | 0.55 |
|  | 8^th^ week | 4.86±1.68 | 4.83±2.27 | 0.91 |
|  | WBC (10^9^/L) |  |  |  |
|  | 0 weeks | 6.96±2.51 | 6.82±2.73 | 0.70 |
|  | 2^nd^ week | 7.23±2.62 | 7.01±2.82 | 0.41 |
|  | 8^th^ week | 7.11±2.71 | 7.19±2.64 | 0.83 |
|  | PLT (10^9^/L) |  |  |  |
|  | 0 weeks | 206.96±82.51 | 216.82±88.73 | 0.41 |
|  | 2^nd^ week | 215.23±78.62 | 207.01±95.82 | 0.50 |
|  | 8^th^ week | 207.11±91.71 | 217.19±86.64 | 0.42 |
| Liver function | ALT (U/L) |  |  |  |
|  | 0 weeks | 31.32±12.07 | 34.29±11.63 | 0.07 |
|  | 2^nd^ week | 34.17±10.85 | 31.83±12.95 | 0.16 |
|  | 8^th^ week | 32.64±13.16 | 32.80±13.17 | 0.93 |
|  | AST (U/L) |  |  |  |
|  | 0 weeks | 30.32±13.12 | 31.29±13.62 | 0.60 |
|  | 2^nd^ week | 31.17±11.84 | 31.83±12.85 | 0.70 |
|  | 8^th^ week | 32.64±12.32 | 30.80±14.13 | 0.32 |
|  | ALP (U/L) |  |  |  |
|  | 0 weeks | 84.34±48.21 | 87.82±51.73 | 0.62 |
|  | 2^nd^ week | 86.53±51.07 | 82.62±52.31 | 0.59 |
|  | 8^th^ week | 84.91±50.76 | 85.63±49.42 | 0.92 |
|  | TP (g/L) |  |  |  |
|  | 0 weeks | 62.17±11.23 | 61.79±12.01 | 0.81 |
|  | 2^nd^ week | 64.72±10.74 | 64.92±11.92 | 0.90 |
|  | 8^th^ week | 65.05±10.38 | 63.81±12.17 | 0.43 |
|  | ALB (g/L) |  |  |  |
|  | 0 weeks | 48.54±21.32 | 47.79±23.01 | 0.81 |
|  | 2^nd^ week | 44.77±25.51 | 49.97±19.91 | 0.10 |
|  | 8^th^ week | 47.05±23.56 | 46.83±24.54 | 0.95 |
|  | GLB (g/L) |  |  |  |
|  | 0 weeks | 25.32±8.23 | 26.79±10.32 | 0.26 |
|  | 2^nd^ week | 24.77±9.65 | 24.97±11.58 | 0.89 |
|  | 8^th^ week | 23.05±7.53 | 23.83±12.72 | 0.59 |
| Kidney function | Scr (umol/L) |  |  |  |
|  | 0 weeks | 72.38±41.11 | 74.57±38.94 | 0.69 |
|  | 2^nd^ week | 75.16±38.23 | 73.63±45.28 | 0.79 |
|  | 8^th^ week | 74.72±40.39 | 72.84±42.64 | 0.75 |
|  | BUN (mmol/L) |  |  |  |
|  | 0 weeks | 5.64±2.81 | 5.79±2.32 | 0.68 |
|  | 2^nd^ week | 5.42±2.74 | 5.95±1.97 | 0.11 |
|  | 8^th^ week | 5.73±2.35 | 5.83±2.13 | 0.75 |
|  | UA (umol/L) |  |  |  |
|  | 0 weeks | 212.54±76.80 | 214.79±82.01 | 0.84 |
|  | 2^nd^ week | 223.77±78.56 | 224.97±79.98 | 0.91 |
|  | 8^th^ week | 205.05±80.21 | 218.83±77.13 | 0.21 |
